# Method of Hydration for Infants Admitted With Bronchiolitis: Physician or Parental Choice?

**DOI:** 10.7759/cureus.13896

**Published:** 2021-03-15

**Authors:** Saima Saqib, Gerald Mugford, Kevin Chan, Robert Porter

**Affiliations:** 1 Faculty of Medicine, Memorial University of Newfoundland, St. John's, CAN; 2 Community Health and Humanities, Memorial University of Newfoundland, St. John's, CAN; 3 Pediatrics, University of Toronto, Toronto, CAN; 4 Pediatrics, Memorial University of Newfoundland, St. John's, CAN

**Keywords:** respiratory bronchiolitis-interstitial lung disease, nasogastric tube (ngt), general pediatrics, acute pain

## Abstract

Objectives

This study examines the practice patterns with respect to the technique of non-oral hydration of infants admitted with bronchiolitis at one Canadian tertiary care institution. Additionally, the authors assess the infants’ parents’ attitudes regarding hydration through a nasogastric (NG) tube instead of an intravenous (IV) line.

Methods

A retrospective chart review was conducted for all infants admitted with bronchiolitis from May 1, 2016, to April 30, 2018, with a focus on the method of hydration, investigation with chest radiography, and use of IV antibiotics. Parents of infants who received IV fluids during the admission were surveyed by mail to assess their perceptions surrounding their child's experience with IV fluid therapy as well as their attitudes toward NG hydration, particularly in cases of difficult IV access.

Results

Of the 101 hospitalized infants, 54 received IV fluids and four received NG fluids. Of the 54 eligible for the survey, 17 completed surveys were returned. Parents were likely to consider NG hydration if suggested by their pediatrician. The proportion was extremely or very likely to consider this intervention increased from 29% in a generic situation to 53% in a scenario where there was more than one unsuccessful IV attempt (p=0.03).

Conclusions

In the institution studied, NG hydration was rarely used. Parents seemed receptive to the idea of NG hydration as an alternative, particularly when IV access is difficult.

## Introduction

Bronchiolitis is a common acute respiratory illness affecting infants and children less than two years of age [1-3]. Usually, the illness is mild; however, hospitalization may be necessary in cases of severe respiratory distress, inability to maintain oxygenation and ventilation, or inadequate oral fluid intake [4,5]. If oral intake is ineffective or unadvisable, supplementary fluids can be administered either through an intravenous (IV) line or via a nasogastric (NG) tube (or, in some cases, an orogastric tube) [6,7]. An electrolyte solution with glucose is typically given if an IV is used, whereas electrolyte solutions, breast milk, or formula can be administered by NG tube.

Clinical trials have suggested that outcomes are similar for IV and NG hydration [8,9]. Clinical practice guidelines recommend either route for hydration [10-12], although at least one guideline favors NG hydration [13]. Practice patterns vary greatly when it comes to the method of hydration, with North American settings typically choosing the IV route [2], while other settings, such as Australia and New Zealand, commonly utilize the NG route, and variation in practice may be determined more by medical culture than evidence or parental preference [14].

Our study aimed to determine local practice patterns around methods of hydration in bronchiolitis, as well as chest radiography and IV antibiotic use, which might indirectly affect the choice of method of hydration, in a cohort of infants admitted with bronchiolitis. We also explored the perceptions of the parents of the infants who received IV fluids regarding the alternative of NG fluid administration.

We hypothesized that NG feeding would be rarely used, but that parents would consider NG hydration as an alternative, especially in cases of difficult IV access.

## Materials and methods

A retrospective chart review of infants less than one year old admitted with bronchiolitis over a two-year period to the Janeway Children’s Health and Rehabilitation Centre, a tertiary children’s hospital in St. John’s, Newfoundland, Canada, was conducted. Subsequently, a mail-out survey was sent to parents of children who received IV fluids. A two-year period was believed adequate to provide a picture of overall practice patterns by a variety of pediatricians in the institution and reduce bias related to individual practice variation.

For the chart review, eligibility criteria were: admission between May 1, 2016, and April 30, 2018; age less than one year at the time of admission; discharge diagnosis of bronchiolitis *or* discharge diagnosis of asthma with nasopharyngeal (NP) swab positive for respiratory syncytial virus or human metapneumovirus *or* discharge diagnosis of pneumonia with wheeze on physical examination and a negative or non-diagnostic chest radiograph. Cases were identified by querying the hospital information system and cross-checking with positive NP swabs during the period to identify missed cases. Cases were excluded if they expired from the index diagnosis or another cause, or if they were intubated during their admission.

Outcomes were determined through a review of the electronic medical record, consisting in part of scanned hand-written notes. The primary outcome for the chart review was the proportion of cases treated with NG hydration. Secondary outcomes included proportions receiving IV hydration, undergoing multiple attempts at IV placement, experiencing local complication at the IV line site, receiving IV antibiotics, having chest radiography performed, and administered supplemental oxygen during the hospital stay, as well as the duration of IV placement.

For the second part of the study, a survey was developed for parents of children who received IV fluids during their admission. Feedback on survey questions was received from a nurse not involved with the study who was also a parent of a young child. The survey consisted of a short introduction to NG hydration with an illustration of a young child with an NG tube in place, followed by questions concerning whether the IV had been successfully placed on the first attempt and whether it was at any point replaced. This was followed by a series of questions rated on a five-point Likert scale concerning the parent’s perception of the child’s distress during the first IV insertion, the parent’s likelihood to consider a recommendation from their pediatrician for NG feeding instead of IV treatment (in a general situation and if there was more than one unsuccessful attempt at IV access), and questions concerning the importance of the following factors in choosing a method of hydration: nutritional value of fluids; discomfort due to insertion of the device (IV or NG); discomfort due to ongoing presence of the device; and success rate of first insertion attempt. At the end of these questions, parents were allowed to express any other comments they wished on the topic. The primary outcome was the proportion of parents who would consider NG hydration as an alternative to IV hydration if suggested by their treating pediatrician in a situation with more than one unsuccessful attempt at IV placement.

In June 2018, questionnaires were mailed to parents identified from the chart review, along with a stamped envelope addressed to the researchers. If there was no response at four weeks, a reminder was mailed, with a second copy of the survey and a stamped envelope.

The identity of patients and parents was protected through the use of study codes and secure handling of paper and electronic documents. Statistical analysis was done using IBM Statistical Package for Social Sciences (SPSS) Version 24 (IBM Corp., Armonk, NY). The descriptive analysis of categorical data was done by using numbers and percentages, and for continuous data by using mean, median, standard deviation, and interquartile range (IQR). Fisher's exact test was used to compare two proportions. 

## Results

Chart review

**Figure 1:**
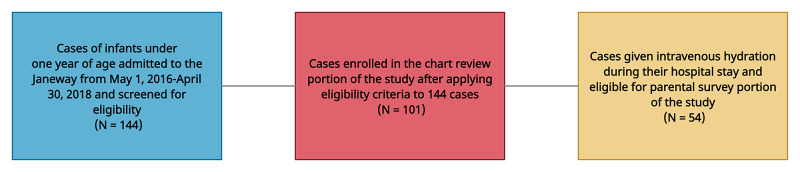
Selection of cases for chart review and parental survey

We screened 144 cases and 101 met eligibility criteria for the chart review. All but one had a discharge diagnosis of bronchiolitis. Of eligible cases, 54 received IV fluids and were therefore eligible for the parental survey (Figure 1). There were two cases with an IV placed who did not receive IV hydration, and they were deemed ineligible for the survey.

Table 1 shows the characteristics of the cases eligible for the chart review. The age ranged from six days to 341 days and the majority were admitted through the emergency department. There were 90 cases admitted to the ward and 11 to the pediatric intensive care unit.

**Table 1:** Baseline data for infants admitted with bronchiolitis NP, nasopharyngeal; CTAS, Canadian Triage and Acuity Scale; PICU, pediatric intensive care unit

Characteristic	Value
Discharge diagnosis: n (%)	
Bronchiolitis	100 (99)
Asthma with positive NP swab	1 (1)
Gender: male n (%)	66 (65.3)
Age in days: mean (SD)	129 (93.8)
Weight in kilograms: mean (SD)	6.2 (2.2)
Route of admission: n (%)	
Emergency department	91 (90.1)
Direct to inpatient location	10 (9.9)
Co-morbidities: n (%)	9 (8.9)
Triage code (n=89): n (%)	
CTAS 2	59 (66.3)
CTAS 3	30 (33.7)
Initial percentage oxygen saturation in room air (n=98): mean (SD)	96 (3.6)
Initial inpatient location: n (%)	
Ward	90 (89.1)
PICU	11 (10.9)

Table 2 shows a summary of the outcomes of interest. Four of the cases received NG fluids, including two who received breast milk and two who received formula.

**Table 2:** Outcomes for chart review of infants admitted with bronchiolitis NG, nasogastric; IV, intravenous; IQR, interquartile range ^a^Of 42 cases for whom relevant documentation was available. ^b^Of 50 cases for whom relevant documentation was available.

Characteristic	Value
NG fluids administered: n (%)	4 (4.0)
Type of fluid through NG tube (n=4): n (%)	
Breast milk	2 (50.0)
Formula	2 (50.0)
IV line placed at study hospital: n (%)	54 (53.5)
IV in place at presentation to study hospital: n (%)	2 (2.0)
Supplementary oxygen administered: n (%)	54 (53.5)
Chest radiography performed: n (%)	74 (73.3)
Received IV fluids (eligible for parental survey): n (%)	54 (53.5)
Number of attempts before successful IV placement^a^: n (%)	
1	28 (66.7)
2	5 (11.9)
3	2 (4.8)
4	3 (7.1)
≥ 5	4 (9.5)
IV line replaced^b^: n (%)	18 (36)
Number of times IV line replaced (n=18): n (%)	
1	16 (88.9)
2	1 (5.6)
4	1 (5.6)
Local IV site complications (swelling, edema, infiltration): n (%)	11 (20.4)
IV medication administered (n=27): n (%)	
Antibiotic	26 (96.3)
Steroid	2 (7.4)
Duration of IV placement in hours: median (IQR)	48 (32.2, 72.5)

To assess morbidity associated with IV treatment, we examined details surrounding its use, including complications, number of insertion attempts, replacement, and duration for those eligible for the survey. Of the 42 cases where relevant nursing notes were available, there was a success on the first attempt in about two-thirds of cases. Replacement of the IV was required in 18 (36%) of the 50 cases for whom nursing progress notes were available, most commonly once during the admission.

Chest radiography was performed in 74 cases, including 25 of 26 survey-eligible cases who received IV antibiotics. When the chest radiographs of these 25 cases were classified, based on final radiologist reports, according to the suggested paradigm of Schuh et al., 27% of cases had a normal study, the majority (65.3%) had findings consistent with bronchiolitis (simple or complex), and a small number (7.7%) had findings inconsistent with typical bronchiolitis [15].

The total duration of IV line placement for 54 cases eligible for the survey was for a median of 48 hours (IQR 32.2, 72.5). One outlier, a seven-month-old infant with multiple comorbidities, had an IV line placed for more than 500 hours.

Parental survey

Of the 54 cases eligible for the parental survey, 33 (61%) were male and the age ranged from six days to 319 days, with a mean of 123 days (SD 106). There were 17 (31%) parental survey responses received (Table 3).

**Table 3:** Outcomes for survey of parents of infants who received intravenous hydration NG, nasogastric; IV, intravenous ^a^IV is defined as intravenous in the introduction to the survey.

n = 17							
Question 1: Was IV^a^ access obtained on the first attempt?							
Response		Yes		No		Don’t recall	
		3		13		1	
Question 2: At any time during your visit did the IV have to be reinserted?							
Response		Yes		No		Don’t recall	
		10		7		0	
Question 3: How would you describe your child’s distress from the insertion of the IV?							
Response	Extreme distress		High distress	Moderate distress	Minimal distress		No distress
	6		5	3	3		0
Question 4: If your pediatrician had offered you the option of nasogastric (NG) feeding INSTEAD of IV treatment, would you consider the option?							
Response	Extremely likely		Very likely	Moderately likely	Somewhat likely		Not likely at all
	4		1	5	4		3
Question 5: If your child had more than one unsuccessful attempt at IV access, would you consider NG feeding as an alternative?							
Response	Extremely likely		Very likely	Moderately likely	Somewhat likely		Not at all
	7		2	4	3		1
Question 6: In choosing a method for providing fluids to an infant who cannot feed for a few days due to a breathing problem, how would you rate the importance of each of the following? 1. Nutrition; 2. Discomfort of insertion; 3. Discomfort due to ongoing presence; 4. Success rate of the first attempt.							
Response	Very important		Important	Undecided	Unimportant		Very unimportant
Nutrition	13		4	0	0		0
Discomfort of insertion	9		6	1	1		0
Discomfort due to ongoing presence	9		6	1	1		0
Success rate of the first attempt	11		6	0	0		0

Of the 37 non-respondents, two were returned to sender as undeliverable. Of the completed surveys, 13 (76%) reported that the IV was not successful on the first attempt and one could not recall. More than half (10 respondents) indicated that the IV was reinserted at some point. About two-thirds (11 respondents) reported that their child’s distress during IV insertion was high or extreme. In general, about a third of parents (five respondents) would be either extremely likely or very likely to consider the option of NG feeds instead of IV fluids if suggested by their pediatrician. This proportion almost doubled (nine respondents) in a hypothetical scenario with more than one unsuccessful IV attempt (p=0.03).

For the questions concerning the importance of various factors when considering a method of hydration, nutritional value and procedural success rate on the first attempt were both considered either very important or important by 100% of respondents. Both discomforts of insertion and due to ongoing presence of the IV or NG tube were considered very important in 53% and important in 35% of parents. The success rate of the first attempt was deemed very important or important by all respondents.

Comments were left by twelve parents (Table 4). A number of them identified issues with IV insertion (including the length of time, number of attempts, number of body sites, and scarring) as well as reinsertion. Two respondents mentioned the need for an IV for antibiotic treatment; one, the skill levels of different providers in IV insertion; and one, the concern of nasal discomfort with an NG tube. Two respondents with previous experience with NG feeding expressed different views, one preferring IV and one NG. One parent who was able to breastfeed during the illness commented that if she was not able to breastfeed, she would prefer NG feeds with breastmilk. One participant highlighted her own distress.

**Table 4:** Written comments from parental surveys NG, nasogastric; IV, intravenous

Parental Comments (n=12)
Always prefer IV over NG, as this patient does not like NG. The overall attitude is much better with IV.
Don’t feel IV was uncomfortable while inserted. Fact that IV had to be re-inserted, causing discomfort again.
I would have chosen whatever methods ensured that my child was fed, I was able to breastfeed, if I wasn’t I would prefer NG feed with breast milk as my first choice.
If antibiotics are required, isn't an IV still needed. NICU nurses were requested for IV, they are more experienced than emerg or ward nurses.
The infant was able to breastfeed while using IV.
It was traumatizing for her and me. It took so long for IV insertion and when it came out, it had to be reinserted.
IV insertion was for IV antibiotics.
IV was inserted after so many attempts (hands, feet, and finally successful on the head, high distress for me and him).
Took 16 attempts to get IV into my 3m old. I definitely would have wanted NG after that experience.
Very difficult to recall, as I myself was distressed and sleep-deprived.
Watching premature twins without any discomfort while on NG more satisfying. IV causes scarring and discomfort.
While it was difficult to deal with IV, I feel I would be hesitant if there was ongoing nasal discomfort for NG.

## Discussion

This study demonstrates that in the institution studied, NG feeding is rarely used in cases of bronchiolitis.

Parental responses suggest they are open to considering NG fluids as an alternative to IV fluids for hydration in cases of bronchiolitis, particularly when IV access is difficult. The study highlights that IV insertion is often very distressing to parents, distress that may not be witnessed by the attending physician. Insertion of an IV line in infants is often not successful on the first attempt and may require many attempts. The two-thirds rate of success on the first attempt on the chart review is an upper limit, and the actual success rate may be significantly lower, as attempts in quick succession may be undocumented. Obtaining informed consent for the procedure should include an acknowledgment of the expected distress, possibility of multiple attempts, and the likelihood of reinsertion (required in more than a third of cases in our sample).

In our study, 27 out of the 54 patients who received IV fluids also received an IV medication, an antibiotic in 26 cases, and a corticosteroid in two cases, making the IV route readily available for hydration. The high proportion treated with antibiotics may have been influenced by overcalling chest radiographs. Chest radiography continues to be commonly performed in infants with bronchiolitis, even though it is not recommended routinely by guidelines. Less than 10% of the cases receiving IV antibiotics in our study had radiographs that were inconsistent with a diagnosis of bronchiolitis. Another rationale for the use of IV antibiotics could have been suspicion of sepsis in very young infants [7,11]. However, of the cases given IV fluids, 13 were less than 28 days old and, of these, only six received IV antibiotics. It is noteworthy that, at the institution studied, there was no local protocol regarding either chest radiography or antibiotic use in patients with bronchiolitis.

Regional variation in the preferred approach to hydration in bronchiolitis could reflect patient preference or medical culture [16,17]. Having both approaches to hydration available would provide meaningful parental choice and an alternative to multiple IV insertions in a child with difficult IV access. This could be facilitated through quality assurance and educational initiatives aimed at facilitating safe NG fluids should parents choose this route [18]. In addition, initiatives to decrease chest radiography would reduce IV antibiotic use and possibly make IV hydration less likely.

This study has a number of limitations, including its retrospective design. As a single-site study, practice patterns and parental expectations might not represent widespread practices and attitudes; however, the two-year period for which data was collected limits bias due to intra-institutional practice variation. While the low usage of NG feeding is consistent with previous research involving sites in North America, the study group was chosen with the strong suspicion that NG feeding would be low, biasing this outcome [18]. Some documentation, particularly nursing notes, was not consistently available and interpretation of handwriting was at times difficult, so some outcome information was missing. We do not expect that missing information was biased.

Our study did not examine the criteria used to determine the need for non-oral hydration or the indications for admission. Although we did not include infants who had an IV line placed but did not receive IV fluids, many of the infants in our study who received IV hydration also received IV medication, and in some cases, IV hydration might have been given as a matter of convenience, as the IV line was already in place due to the perceived need for medication.

The number of participants in the parental surveys was quite low, significantly limiting the interpretation of this data, and response bias is a concern, with parents with negative experiences possibly being more likely to respond. The time between the admission and the survey was variable (up to around two years), likely affecting recall of the details of care as well as contributing to the low response rate. Another limitation of the study is that it only addresses experiences with IV hydration and, aside from the comments section of the survey, provides no information about an experience with NG hydration.

## Conclusions

Based on our limited data, it appears that chest radiography, IV antibiotics, and IV hydration were overused in our study, with significant negative impacts. If non-oral hydration is needed for infants admitted with bronchiolitis, evidence suggests that both IV and NG fluids are viable options. Availability of both options and clear communication of the risks and benefits of each will facilitate meaningful parental input into this aspect of their infants’ care.
